# Outcomes of Resectable Locally Advanced Non-Small Cell Lung Cancer After Neoadjuvant Chemoimmunotherapy: A Single Institution Experience

**DOI:** 10.3390/jcm14030988

**Published:** 2025-02-04

**Authors:** Jose Noy, Alexander Chang, Nelly P. Chow, Javier De Jesus Fernandez, Rohan Dureja, Luis Miguel Cotamo, Ahmed Alnajar, Dao M. Nguyen, Nestor Villamizar

**Affiliations:** Miller School of Medicine, University of Miami, 1475 NW 12th Ave, Miami, FL 33136, USA; jose.noy@jhsmiami.org (J.N.); alexander.chang@jhsmiami.org (A.C.); rxd671@miami.edu (R.D.);

**Keywords:** neoadjuvant chemoimmunotherapy, lung cancer, surgical outcomes, robotic surgery, postoperative complications, pathologic response, survival

## Abstract

**Introduction:** Immunotherapy has revolutionized the treatment for locally advanced resectable non-small-cell lung cancer (NSCLC). In clinical trials, the combination of neoadjuvant immunotherapy and chemotherapy has resulted in a higher rate of pathologic complete response in comparison with neoadjuvant chemotherapy alone. Our study aims to describe surgical and oncological outcomes after neoadjuvant chemoimmunotherapy and lung resection at our academic center outside clinical trials. **Methods:** We retrospectively analyzed 54 patients who received neoadjuvant chemoimmunotherapy and underwent surgical resection from 2018 to 2024. Demographics, pre-operative systemic treatment, surgical approach and postoperative outcomes were evaluated. **Results:** The median age was 65 years, 46% were female, and 67% of patients had a non-squamous histology, chiefly adenocarcinoma. The most common clinical stage was IIIA (54%). Major findings include a 41% pathologic complete response (pCR) and 52% major pathologic response (MPR) rate. Neoadjuvant chemoimmunotherapy resulted in downstaging in 78% (*n* = 42) of patients. Most patients (83%) had their operation completed robotically. R0 resection was achieved in 96%. Median length of stay was significantly shorter after robotic operations, with no significant difference in complications compared to the open group. At a median follow up of 16 months, 24 months of recurrence-free survival was estimated at 76% (95% CI: 61–94) and overall survival, 93% (CI: 84–100). **Conclusion:** At our medical center, induction chemoimmunotherapy followed by anatomic lung resection has resulted in a high rate of complete pathologic response, overall survival and recurrence-free survival. The robotic approach after induction chemoimmunotherapy is safe and associated with shorter length of stay and faster recovery time.

## 1. Introduction

Despite many advances in treatment, lung cancer remains the leading cause of cancer-related deaths in the United States [1]. A predicted 234,850 new cases of lung cancer will be diagnosed in the United States in 2024 and approximately 125,570 deaths will result [2]. It is estimated that 25–30% of non-small cell lung cancer (NSCLC) cases are resectable. However, 5-year survival rates are above 90% for resected stage IA in contrast to below 40% in resected stage IIIA [3]. Recurrence after surgery has been reported to be up to 50% in stage IIIA disease [4]. The addition of neoadjuvant chemotherapy only contributed to 5% absolute difference in 5-year recurrence-free survival and overall survival [5]. Pathologic response rate has been proposed as a surrogate for survival in resected lung cancer. Pathological complete response (pCR) is defined as 0% viable malignant cells in the resected surgical specimen after neoadjuvant therapy, and major pathological response (MPR), defined as ≤10% viable malignant cells noted in the resected primary tumor sample after neoadjuvant therapy [6]. Overall, the median rate of pCR reported from 15 trials of neoadjuvant chemotherapy was 4% (range 0–16%) [7].

Immunotherapy represents a groundbreaking approach in the field of oncology, revolutionizing the treatment of multiple cancers, including NSCLC. In the neoadjuvant setting, immunotherapy provides an early opportunity to treat micro metastatic disease and enhances the immune response when bulk tumor and tumor antigens are still present during the treatment [8,9]. Several landmark studies have examined the efficacy of neoadjuvant immunotherapy, both alone and in combination with other modalities such as radiation therapy and chemotherapy, in the treatment of resectable locally advanced NSCLC. Across multiple phase II/III trials including LCMC3, NADIM, NADIM II, Keynote 671, Checkmate 816 and Aegean, neoadjuvant chemoimmunotherapy led to high rates of pCR and MPR [10,11,12,13,14,15].

Even with the advent of immunotherapy, surgical resection remains a pivotal component in the treatment of resectable locally advanced NSCLC. Patients with limited mediastinal lymph node involvement are generally deemed more favorable surgical candidates than those with multi-station or bulky mediastinal lymphadenopathy. However, there are no specific guidelines to determine precisely which tumors should be considered resectable or not, and it ultimately depends on a multidisciplinary discussion involving lung cancer experts, patients and their families with individual assessment of risks and benefits [16].

Our multidisciplinary team at the University of Miami Health system has embraced early implementation of new strategies when preliminary results of ongoing clinical trials are promising. The first robotic lobectomy after chemoimmunotherapy was performed at our institution in February 2018. The purpose of this study is to describe surgical and oncological outcomes after neoadjuvant chemoimmunotherapy and lung resection at our academic center outside clinical trials.

## 2. Methods

The selected timeframe was between February 2018 and August 2024. These patients were identified through a consistently updated database of patients that underwent thoracic surgery at the University of Miami. Electronic medical records of patients who received induction chemotherapy and immunotherapy followed by lung resection were reviewed retrospectively. Patients who completed induction chemoimmunotherapy and then underwent surgical resection were included in this study. Patients who did not complete induction therapy or were not candidates for surgical resection were not included.

The primary objective of this study was to describe the surgical and oncological outcomes after neoadjuvant chemoimmunotherapy and lung resection in our institution. Furthermore, data were contrasted with current available clinical trials evaluating the same outcomes. Main outcomes described included pathological response, complications and surgical approach (open thoracotomy versus robotic surgery). While this study did not establish rigid institutional guidelines for perioperative chemoimmunotherapy, it reflects the real-world application of evolving neoadjuvant strategies in a multidisciplinary setting.

Our multidisciplinary thoracic oncology tumor board at the University of Miami Health System currently favors induction chemoimmunotherapy for any tumor without targetable mutations that are larger than 4 cm, ipsilateral hilar or mediastinal node positive disease, chest wall invasion, or solitary brain metastasis. Only in very select patients has induction chemoimmunotherapy been administered in contralateral mediastinal disease or solitary metastatic disease in sites other than the brain (bone, adrenal). The final decision regarding treatment approach depends on shared decision making between the treating physicians and patients. The 8th edition of the American Joint Committee on Cancer was utilized for staging classification.

Every patient included in this study had a staging PET/CT scan, brain MRI and hilar/mediastinal lymph node biopsy via endobronchial ultrasound with transbronchial needle aspiration (EBUS-TBNA) before induction therapy. Every patient had a restaging PET/CT scan after completion of induction therapy. However, restaging brain MRI and EBUS-TBNA were not routinely performed. The chemoimmunotherapy regimen was up to the discretion of the treating oncologist. The choice of specific drugs and number of cycles was influenced by evolving clinical trial data and institutional practices. Our multidisciplinary tumor board recommended one year of adjuvant immunotherapy for all patients regardless of the pathologic response at the time of surgery.

Surgical resections were performed by the institution’s two primary thoracic surgeons and the approach method was up to the discretion of the surgeon. Histopathologic assessment of resection specimens for degree of pathologic response was performed according to the recommendations from William et al. and the International Association of Lung Cancer (IASLC). Major pathologic response (MPR) was defined as less than 10% residual tumor across all histologic subtypes of lung cancer in the totality of the resected lung specimen. Classification of pCR was determined if no residual tumor was present in the resected lung specimen or excised lymph nodes. While William et al. did not establish guidelines for classification of specimens with greater than 10% residual tumor, several studies have defined this as incomplete pathologic response (IPR) [12,17].

Continuous variables were described as medians (interquartile range [IQR]) after normality assessment with Shapiro–Wilk test and histograms, and categorical variables were summarized by counts (with percentages). Continuous value comparisons were conducted using the Wilcoxon rank sum test, while categorical value comparisons were assessed using Pearson’s Chi-squared test or Fisher’s exact test, as applicable. The analysis was performed using R version 4.3.1 (2023-06-16 ucrt, R Foundation for Statistical Computing, Vienna, Austria).

## 3. Results

A total of 54 patient charts were reviewed from February 2018 to August 2024. The demographic and clinical characteristics of the cohort are summarized in Table 1. The median age of patients was 65 years (range, 50–85) with a similar sex distribution of 25 female patients (46%) and 29 male patients (54%). Most of the patients were white (*n* = 49, 91%) with a majority identifying their ethnicity as Hispanic or Latino (*n* = 34, 63%). Most patients were former smokers (*n* = 41, 76%) and median FEV1 and DLCO were 84% and 68% [(IQR 54–127 and 33–129)], respectively. A single patient with a DLCO of 33% that underwent a left pneumonectomy had negligible perfusion to the left lung. Functional status, assessed with the ECOG performance score, was either an ECOG of 0 (56%) or 1 (43%) with one patient having an ECOG score of 2.

Tumor characteristics are listed in Table 2. Most of the patients had clinical stage IIIA disease (*n* = 29, 54%) followed by stage II disease (*n* = 15, 28%), stage IIIB disease (*n* = 5, 9%), or oligometastatic stage IV disease (*n* = 5, 9%). Four of the five patients with stage IV had a single brain lesion that was treated with gamma knife radiosurgery prior to induction therapy. The other patient with clinical stage IV disease had questionable bone lesions in the thoracic spine that were never biopsied or evaluated with MRI. These lesions disappeared on follow up imaging after induction therapy and tumor board recommendation was to proceed with surgery. Within the clinical staging there was heterogeneity with the TNM staging of the patients. A total of 18 patients had T1 disease (33%); 11 patients had T2 disease (20%); 16 patients had T3 disease (30%); and 9 patients had T4 disease (17%). In total, 32 patients (59%) had clinically positive lymph nodes with a majority of these involving an N2 station (*n* = 25). In total, 80% (*n* = 20) of patients with N2 disease were positive only at a single station. The right upper lobe was the common location for the primary tumor (*n* = 21, 39%). The most common histologic subtype was adenocarcinoma (*n =* 36, 67%), with the remainder being squamous cell carcinoma (*n=* 18, 33%). PD-L1 expression was variable with most tumors having <1% PD-L1 expression (*n* = 26, 48%).

Systemic treatment characteristics are summarized in Table 3. The most prescribed neoadjuvant immunotherapy drug was nivolumab (*n =* 28, 52%), followed by pembrolizumab (*n =* 24, 44%). Two patients who had neoadjuvant chemoimmunotherapy at outside facilities did not have their regimens available. Before FDA approval of nivolumab in March 2022, 15 patients had induction chemoimmunotherapy. Eleven patients received pembrolizumab, three patients received nivolumab and one patient was treated at an outside institution with an unknown regimen. Overall, 74% of all patients on the study had Next Generation Sequencing (NGS) prior to induction chemotherapy and 80% of patients with adenocarcinoma had NGS before induction therapy. In our cohort, 17.5% of the patients with NGS had a targetable mutation, unrelated to PDL1 status. One patient was positive for EGFR and one positive for ALK. The mutations were both discovered postoperatively on final surgical pathology. The patient with the ALK mutation had pre-induction NGS performed on the staging EBUS but the samples were insufficient for analysis. The patient with the EGFR mutation only had pre-induction blood NGS testing that did not identify any targetable mutations.

A vast majority of patients had their surgery completed with a robotic approach (*n* = 45, 83%) with the remainder having the surgery completed open (*n* = 9, 17%). Four of these open resections were pneumonectomy, 2 were bilobectomy, 1 was a right lower lobectomy, 3 were lobectomy with en-bloc chest wall resection, 1 of those was a conversion from a robotic approach due to hemorrhage from avulsion of a posterior ascending branch of the pulmonary artery while dissecting the right upper lobe bronchus. The most common robotic resection was a single lobectomy (*n* = 34) but other complex procedures were also completed robotically including pneumonectomies (*n* = 2), en-bloc chest wall resections (*n* = 5, including 2 Pancoast tumors) and sleeve lobectomies (*n* = 2).

Surgical outcomes are summarized in Table 4. On final pathology, almost all patients had an R0 resection (*n* = 52, 96%). Only two patients (4%) had an R1 resection with no patients having an R2 resection. In total, 38 (70%) patients had no complications. Of the 16 patients who had complications, there were no patients who had peri-operative myocardial infarction, pulmonary embolism, hemorrhage or death. Five patients had prolonged air leak (defined as more than 5 days); five patients had atelectasis that required bronchoscopy; four patients developed post-operative atrial fibrillation. Three patients had post-operative chylothorax and two of these patients required a return to the operating room for operative ligation of the thoracic duct. No other patients required a return to the operating room. 

Complications within the robotic and open approaches were also detailed (Table 5). There were no differences in complications between the open and robotic groups (22% vs. 29%, *p* > 0.9). There were also no significant differences in air leak, atelectasis, pleural effusion requiring procedural drainage, respiratory failure requiring mechanical ventilation, pneumonia, atrial fibrillation, hemorrhage, chylothorax, readmission, or return to the operating room. The only significant difference was length of stay which was longer in the open group compared to the robotic. The median length of stay for those who had an open operation was 5 days (IQR 4–7), whereas those who had a minimally invasive operation had a median length of stay of 3 days (IQR 2–5). Complications encountered that increased length of stay for robotic cases included persistent air leaks, atelectasis and chylothorax. Ten patients (19%) were discharged home on postoperative day 1 after a robotic lobectomy. The median time for a robotic lobectomy after induction chemoimmunotherapy was 240.5 min (IQR: 182.0–299.25). There was a single robotic sleeve lobectomy which took 669 min. The median time for robotic pneumonectomy was 331 min (IQR: 270.5–372.5). The median time for robotic Pancoast tumor was 531 min (IQR: 437.0–625.0). In comparison, the median time for open surgery was 336 min (IQR: 332.0–337.0).

**Figure 1:**
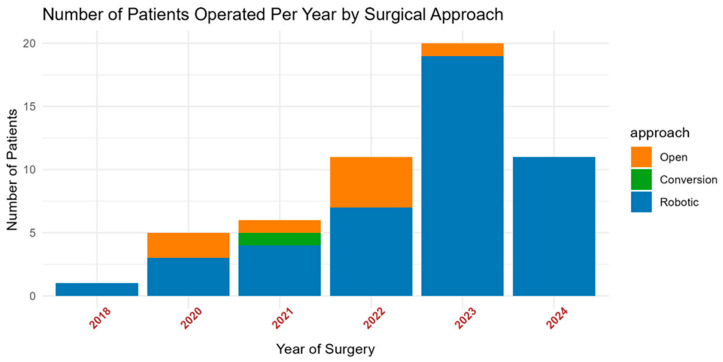
Number of cases stratified by year.

Figure 1 highlights the change over time of cases that were performed at the UHealth Tower. The first case after induction therapy was performed in 2018 with an increase in the number of cases each year. The open approach was more commonly performed early in our experience with these cases. FDA approval of nivolumab was in March of 2022, followed by pembrolizumab in October of 2023.

**Figure 2:**
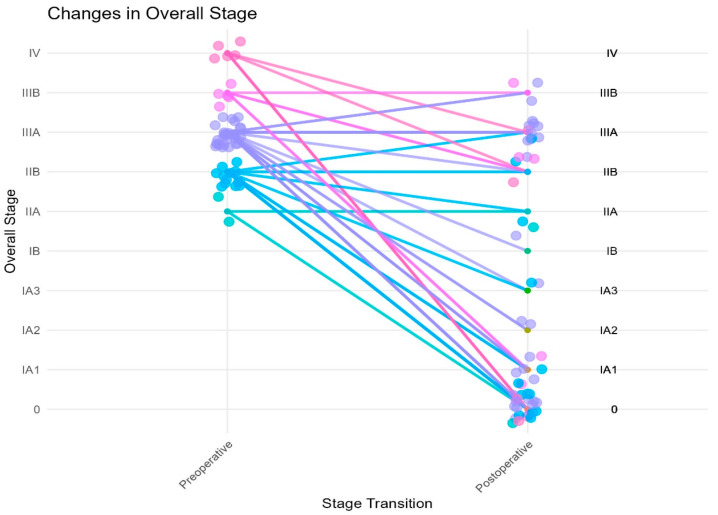
Preoperative versus postoperative stage.

Downstaging occurred in 42 (78%) of our patients after induction chemoimmunotherapy based on their clinical versus pathologic staging (Figure 2). There were two patients who were clinical stage IIA; one (50%) had pCR and the other remained pathologic stage IIA. Of the 13 patients who were clinical stage IIB, 7 (54%) patients had pCR, 2 were downstaged to pathologic stage IA1, 1 to stage IA3, 1 to stage IIA, 1 remained stage IIB and 1 was upstaged to IIIA. There were 29 patients clinical stage IIIA; 11 (38%) had pCR, 4 were downstaged to pathologic stage IA1, 2 to IA2, 1 to IA3, 1 to IB and 1 to IIB. There were seven patients clinical stage IIIA that remained pathologic stage IIIA and two patients who were upstaged to IIIB. There were five patients clinical stage IIIB; two were downstaged to pathologic stage IA1, two to stage IIB and one remained pathologic stage IIIB. There were five patients clinical stage IV; after treatment, three (60%) of these patients had pCR, one was downstaged to pathologic stage IIB and one to stage IIIA.

Five patients had multi-station mediastinal lymph node disease before induction therapy. One of them had pCR after induction therapy, two were downstaged to IA1, one to IA3 and one to IIB. Two patients were noted to have T upstaging postoperatively: one patient had visceral pleural invasion on final pathology and the other had an increase in the primary tumor from T3 (5.9 cm) to T4 (7.1 cm).

**Figure 3:**
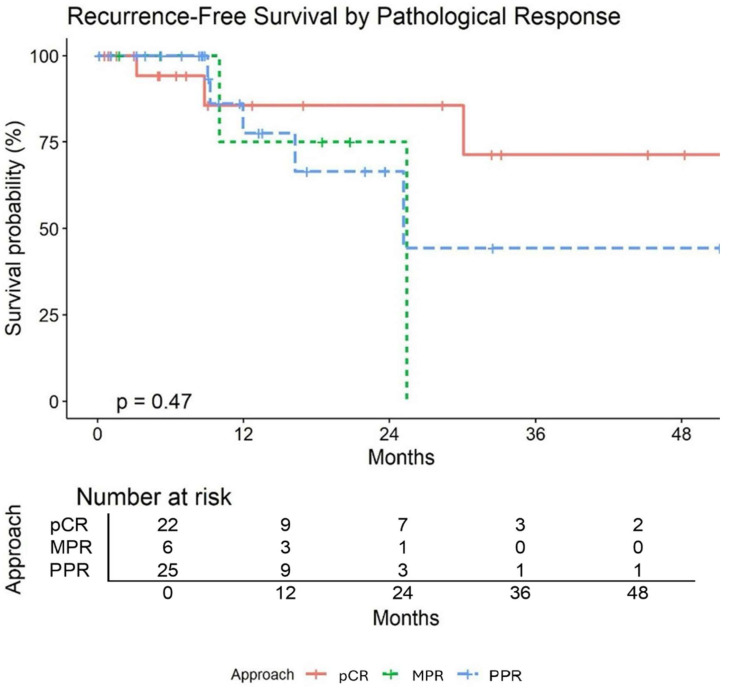
Recurrence-free survival by pathological response.

Table 6 summarizes pathological and survival outcomes. After surgical resection, pCR was achieved in 22 patients (41%), MPR was achieved in 28 patients (52%), and IPR was achieved in 26 patients (48%). The median follow up time was 16.08 months (95% CI: 14–18.50). Three of the 54 patients died (94% survival). Of the patients who died, two of them had pCR and the other had MPR. One patient died of an acute myeloid leukemic crisis that was felt to be unrelated to treatment. Nine patients experienced recurrence (17%). Two patients had locoregional recurrence: one patient had recurrence at a staple line and the other had recurrence at a station 7 lymph node. The remaining seven patients had distant recurrence to various locations (contralateral lung, liver, bone, brain). Of those with distant recurrence two of them had pCR. Recurrence-free survival (RFS) at 24 months was estimated as 76% (95% CI: 61–94). pCR was associated with higher RFS; however, the association was not statistically significant (Figure 3).

## 4. Discussion

Locally advanced NSCLC requires a multidisciplinary approach to management due to the high risk of relapse in these patients. From a surgical perspective, a primary consideration is resectability of the patient’s disease. Traditional contraindications for surgical resection have included the presence of contralateral mediastinal or ipsilateral supraclavicular lymphadenopathy (N3), multi-station N2 disease, bulky N2 disease (mediastinal lymph nodes with a short-axis diameter over 3 cm or extra-nodal extension into adjacent tissue structures) or distant metastasis [18]. However, dramatic radiologic and pathologic response to neoadjuvant chemoimmunotherapy has promoted the enthusiasm for surgical consideration of those patients, particularly if they are young with good performance status. Neoadjuvant chemoimmunotherapy has thus emerged as a promising strategy in the treatment of locally advanced NSCLC, improving outcomes through tumor downstaging, enhancing resectability and improving survival [19]. Our retrospective study, in alignment with findings from prior landmark trials, underscores the efficacy of neoadjuvant chemoimmunotherapy in this clinical context. It also highlights the feasibility and safety of minimally invasive robotic surgery in the post induction setting. All cases of locoregional-advanced and oligometastatic NSCLC are presented at a weekly thoracic tumor board conference at the Sylvester Comprehensive Cancer Center, University of Miami Health System. Once a patient with locally advanced NSCLC is deemed “resectable” and no actionable mutations are found, the pathway towards neoadjuvant chemoimmunotherapy is offered.

Our facility initially began offering induction chemoimmunotherapy in 2017 and performed the first operation on a patient who received induction therapy in 2/2018. This is around the time that several landmark trials (NADIM, KEYNOTE, CHECKMATE all in 2017) were enrolling patients. With FDA approval of nivolumab in 2022 and pembrolizumab in 2023 in the neoadjuvant setting, we saw an increase in the number of patients who underwent surgical resection after induction chemoimmunotherapy. In these patients, we noted a significant percentage of patients who achieved complete and major pathologic response. The percentage of patients with pCR and MPR piqued our interest as it appeared to be higher (not statistically significant) than those in the landmark studies (Table 7). This triggered interest in which patient factors could contribute to a greater pathologic response. MPR and pCR have been strongly associated with event-free survival (EFS) and overall survival (OS) in patients with resectable NSCLC in the setting of neoadjuvant chemoimmunotherapy [7,9,10,11,12,13,17,20,21,22]. Miami is home to a large population who identify as Hispanic or Latino. It should be mentioned that most patients in our study identified as White (*n* = 49, 91%), with 34 of the patients (63%) identifying as Hispanic or Latino. Our data should be interpreted with this in mind as our patient population may not represent the demographics/patients that other centers treat. There may be a genetic component contributing to the higher rate of pathologic response in our study that would need further investigation. Additionally, the median BMI of this study was 26.1 kg/m^2^, with 52% of patients not having a listed co-morbidity in our study (*n* = 28). Patients with systemic diseases such as diabetes or obesity could have a less robust immune response, with induction chemoimmunotherapy leading to worse outcomes. However, on statistical analysis, there was no significant difference in patients with diabetes and/or obesity and pathological response. Our sample size is likely too small to have a meaningful comparison, and a larger population would be necessary to investigate this further.

Other factors that could account for our higher rate of pathologic response could be gender or tumor histology. This study had relatively even representation of male and female patients (*n* = 29, 54% versus *n* = 25, 46%) which contrasts other trials who had a greater predominance of male patients compared to female patients. Additionally, we had a larger number of patients whose pathology was non-squamous histology compared to those with a squamous histology (67% versus 33%). The histologic distribution in our study is similar to NADIM II trial which had 63.2% non-squamous histology and 36.8% squamous histology. pCR of 37% in NADIM II trial compares similary to pCR in our study. It is also possible that a specific chemoimmunotherapy regimen may result in a higher pCR. The most common regimen in our study was Carboplatin, Pemetrexed and Pembrolizumab. It is clear in AEGEAN and other trials such as CheckMate 816 that greater PD-L1 expression has a significant impact on pCR. In our study, we did not find PD-L1 expression to predict pCR, likely related to our small sample size. The vast majority of our patients experienced downstaging after induction chemoimmunotherapy with similar impact on T stage and N stage. Significant downstaging included patients with stage IV disease with a single metastatic site and stage III disease with multi-station mediastinal lymph node involvement.

Only 30% of our patients had a postoperative complication, which is lower compared to other studies (not statistically significant). However, in comparison to our own institution’s database of patients, this complication rate is higher than patients undergoing anatomic lung resection without induction chemoimmunotherapy. We implemented an enhanced recovery after thoracic surgery around the same time as the first robotic lobectomy after induction chemoimmunotherapy in February of 2018. We have performed 741 anatomic lung resections since then including 54 patients of this study. Approximately, 15% of all patients have experienced a complication. It may be inferred from this comparison that patients who undergo induction chemoimmunotherapy have a higher overall risk for perioperative complications compared to the general thoracic surgical population. However, surgery in these patients can still be safely achieved using the robotic platform. The use of minimally invasive surgery in our patient population was more common than in reported clinical trials (Table 7). We had a low overall number of open cases (9, 17%). Eight of these cases were planned as an open approach, with many of these being earlier in our data set. As we became more comfortable in performing robotic surgery in post induction patients, no patient since January 2023 has undergone an open surgical resection. Similarly, the one robotic case that was converted to open for pulmonary artery bleeding was relatively early in our experience with these cases. Post-operative characteristics were analyzed between the robotic and open group. The only significant difference between these groups is that the open group had a longer length of stay than the robotic group (5 days vs. 3 days). There was no significant difference for any complication, including air leak, pleural effusion, respiratory failure, pneumonia, atelectasis, pulmonary embolism, arrhythmia, chylothorax or return to the operating room. There were no mortalities within the first 60 days of an operation. These findings highlight that robotic surgery resulted in a shorter postoperative recovery period and could safely be performed in this patient population without increase in postoperative complications. Furthermore, robotic surgery was associated with sound oncologic outcomes as 96% of patients in our study had an R0 resection, 4% had an R1 resection and no patient had an R2 resection.

During the study period, there were different neoadjuvant regimens utilized as new data regarding the use of perioperative chemoimmunotherapy in the treatment of NSCLC emerged from the following trials: KEYNOTE-671, CheckMate816, NADIM and NADIM II [11,12,13,23]. In our single institution experience, the majority of patients received neoadjuvant chemoimmunotherapy with four cycles of platinum-based chemotherapy plus pembrolizumab followed by adjuvant pembrolizumab (replicating the regimen of Keynote-671) for one year regardless of the pathologic response at the time of surgery [23]. Once a neoadjuvant chemoimmunotherapy regimen with nivolumab was approved as per the CheckMate 816 trial, there was a trend toward patients receiving three instead of four cycles of induction chemoimmunotherapy with no apparent negative impact in the rate of pathologic responses [13]. Overall, patients completed the planned courses of neoadjuvant chemoimmunotherapy involving either three or four cycles of therapy with good tolerance. In our study, all patients completed at least three cycles of neoadjuvant therapy prior to proceeding with surgical resection. However, this does not encompass patients who may not have made it to surgical resection due to intolerance of systemic therapy. Adjuvant therapy was administered to patients in accordance with the IMpower 010 regimen and at the treating physician’s discretion [24].

Median follow up time in our patient population was 16.08 months (95% CI: 14–18.50). Event free survival (EFS) at 24 months was estimated at 76% (95% CI: 61–94). Overall survival (OS) at 24 months was estimated at 93% (95% CI: 84–100). These results compare favorably to EFS and OS in RCTs (Table 7).

There are several limitations to this study which include its retrospective nature, single institution experience and small sample size that is likely subject to selection bias. This study does not capture the percentage of patients who progressed during induction therapy and never underwent an operation. The median follow up in our study of only 16.08 months limits our ability to establish comparisons of EFS and OS with CheckMate 816 and KeyNote 671. At the time results were published, those trials had a longer median follow up of 29.5 months and 25.2 months, respectively.

## 5. Conclusions

In our patient population induction chemoimmunotherapy followed by lung resection has resulted in a good pathologic response rate. Most patients can undergo robotic operations safely with shorter length of stay and faster recovery time. A collaborative patient-centered multidisciplinary approach remains the keystone to improving outcomes in these complex cases.

## Figures and Tables

**Table 1:** Demographic characteristics *n* = 54.

Age (Years) Median [IQR]	65 [50–80]
<65 y *n* (%)	26 (48%)
≥65 y *n* (%)	28 (52%)
**Sex *n* (%)**	
Male	29 (54%)
Female	25 (46%)
**Ethnicity *n* (%)**	
Hispanic or Latino	34 (63%)
Non-Hispanic or Latino	18 (33%)
Unknown	2 (4%)
**Race *n* (%)**	
White	49 (91%)
Black or African	2 (4%)
Asian	1 (2%)
Other	2 (4%)
**Body Mass Index Median [IQR]**	26.1 [18.4–40.2]
>26 *n* (%)	27 (50%)
≤26 *n* (%)	27 (50%)
**History of tobacco use *n* (%)**	
Never smoked	9 (17%)
Former smoked	41 (76%)
Current smoker	4 (7%)
**ECOC performance status score *n* (%)**	
0	30 (56%)
1	23 (43%)
2	1 (2%)
**Comorbidities *n* (%)**	
Any Comorbidity	26 (48%)
No Comorbidity	28 (52%)
COPD	8 (15%)
Coronary Artery Disease	6 (11%)
Diabetes Mellitus	13 (24%)
Rheumatoid Arthritis	1 (2%)
Tuberculosis	0 (0%)
History of Histoplasmosis	0 (0%)
Previous Thoracic Surgery (Ipsilateral)	4 (7%)
**Pulmonary Function**	
FEV1 (%) Median [IQR]	84 [54–127]
DLCO (%) Median [IQR]	68 [33–129]

**Table 2:** Tumor characteristics *n* = 54.

Location *n* (%)	
Right Upper Lobe	21 (39%)
Right Middle Lobe	3 (6%)
Right Lower Lobe	12 (22%)
Left Upper Lobe	10 (19%)
Left Lower Lobe	8 (15%)
**cStage *n* (%)**	
II	15 (28%)
IIIA	29 (54%)
IIIB	5 (9%)
IV	5 (9%)
**cTNM Classification (Primary Tumor) *n* (%)**	
T1	18 (33%)
T2	11 (20%)
T3	16 (30%)
T4	9 (17%)
**cTNM Classification, Regional Lymph Nodes *n* (%)**	
N0	22 (41%)
N1	7 (13%)
N2	25 (46%)
Single station	20 (80%)
Multi-station	5 (20%)
**Histologic Classification *n* (%)**	
Squamous	18 (33%)
Nonsquamous	36 (67%)
**PD-L1 Expression Level *n* (%)**	
<1%	26 (48%)
1–49%	12 (22%)
>50%	16 (30%)

**Table 3:** Treatment characteristics *n* = 54.

Chemotherapy/Immunotherapy Regimen *n* (%)	
Carboplatin-Pemetrexed-Pembrolizumab	17 (31%)
Carboplatin-Paclitaxel-Pembrolizumab	5 (9%)
Carboplatin-Pemetrexed-Nivolumab	8 (15%)
Carboplatin-Paclitaxel-Nivolumab	11 (20%)
Cisplatin-Pemetrexed-Pembrolizumab	2 (4%)
Cisplatin-Paclitaxel-Nivolumab	2 (4%)
Cisplatin-Pemetrexed-Nivolumab	7 (13%)
Unknown (outside UM)	2 (4%)
**Surgical Approach *n* (%)**	
Robotic	45 (83%)
Open surgery	9 (17%)
**Type of Procedure *n* (%)**	
Lobectomy	35 (65%)
Bilobectomy	2 (4%)
Pneumonectomy	6 (11%)
Sleeve Lobectomy	2 (4%)
Lobectomy + Chest Wall Resection	7 (13%)
Segmentectomy	2 (4%)
Bronchoplasty	1 (2%)
Conversion	1(2%)

**Table 4:** Surgical outcomes. *n =* 54.

**Margin *n* (%)**	
R0	52 (96%)
R1	2 (4%)
R2	0 (0%)
**Complications *n* (%)**	
No complication	38 (70%)
One or more complications	16 (30%)
Air Leak	5 (9%)
Atelectasis	5 (9%)
Atrial Fibrillation	4 (7%)
Chylothorax	3 (6%)
Effusion	1 (2%)
Respiratory failure	1 (2%)
Pneumonia	1(2%)
Other	1 (2%)
Myocardial Infarction	0 (0%)
Pulmonary embolism	0 (0%)
Hemorrhage	0 (0%)
**Readmission *n* (%)**	3 (6%)
**Death (60 day) *n* (%)**	0 (0%)
**Length of Stay in days Median [IQR]**	3 (1–25)
**Discharge on POD#1 *n* (%)**	10 (19%)

**Table 5:** Surgical outcomes: open vs. robotic.

Outcome	Overall, *n*= 54	Open Surgery, *n*= 9	Robotic, *n*= 45	*p*-Value *
**Length of Stay (Days) Median [IQR]**	3.0 [2.0, 5.0]	5.0 [4.0, 7.0]	3.0 [2.0, 5.0]	0.01
**Complications, Overall *n* (%)**	15 (28%)	2 (22%)	13 (29%)	>0.9
Air Leak	3 (5.6%)	0 (0%)	3 (6.7%)	>0.9
Atelectasis	5 (9.3%)	1 (11%)	4 (8.9%)	>0.9
Pleural Effusion	1 (1.9%)	0 (0%)	1 (2.2%)	>0.9
Respiratory Failure	1 (1.9%)	0 (0%)	1 (2.2%)	>0.9
Pneumonia	1 (1.9%)	0 (0%)	1 (2.2%)	>0.9
Myocardial Infarction	0 (0%)	0 (0%)	0 (0%)	
Pulmonary Embolism	0 (0%)	0 (0%)	0 (0%)	
Atrial Fibrillation	4 (7.4%)	0 (0%)	4 (8.9%)	>0.9
Hemorrhage	0 (0%)	0 (0%)	0 (0%)	
Chylothorax	3 (5.6%)	1 (11%)	2 (4.4%)	0.4
Readmission to the Hospital	3 (5.6%)	0 (0%)	3 (6.7%)	>0.9
Return to OR	2 (3.7%)	1 (11%)	1 (2.2%)	0.3
Unspecified	1 (1.9%)	0 (0%)	1 (2.2%)	>0.9
**Complication Absolute Number *n* (%)**				
0	39 (72%)	7 (78%)	32 (71%)	0.7
1	10 (19%)	1 (11%)	9 (20%)	
2	2 (3.7%)	1 (11%)	1 (2.2%)	
3	2 (3.7%)	0 (0%)	2 (4.4%)	
4	1 (1.9%)	0 (0%)	1 (2.2%)	

* Wilcoxon rank sum test; Fisher’s exact test.

**Table 6:** Pathological and survival outcomes. *n =* 54.

Pathologic Complete Response *n* (%)	22 (41%)
Major Pathologic Response *n* (%)	28 (52%)
Pathologic response *n* (%)	26 (48%)
Length of Follow Up, months, Median [IQR]	16.08 [14.00–18.50]
Recurrence *n* (%)	9 (17%)
Overall Survival Rate *n* (%)	51 (94%)
24-Month Recurrence-Free Survival *n*(%)	41 (76%)

**Table 7:** Summary of main outcomes of recent trials compared with our institution.

	Our Institution	CheckMate 816 [13]	AEGEAN [15]	KEYNOTE–671 [23]	NADIM 2 [12]
**Chemo-Immunotherapy**	Perioperative	Neoadjuvant	Perioperative	Perioperative	Perioperative
**Immunotherapy Agent**	Choice of Oncologist	Nivolumab	Durvalumab	Pembrolizumab	Nivolumab
**Subjects N**	54	149	295	325	53
**Age Median [IQR]**	65 [50–85]	64 [41–82]	68 [30–88]	63 [26–83]	65 [58–70]
**Gender (M/F; %)**	54/46	71.5/28.5	68.9/31.1	70.3/29.7	63.2/36.8
**Pre-treatment stages (%)**					
IB-II	28	36.3	28.4	29.7	0
IIIA/IIIB	54/9	63.1 (total)	47.3/24	54.7/15.6	73.3/22.8
N2 nodal metastasis	46	-	49	42	72
**Surgical data (%)**					
MITS	83	30	50	-	-
Conversion MITS to open	2	11.4	-	-	-
R0	96	83.2	94.7	92	92.3
R1	4	10.7	4	5.2	1.9
R2	0	3.4	0.9	1.2	0
90-day operative mortality	0	3.1	-	4	1.9
Any complication	30	41.6	40	71.1	-
**Pathology details (%)**					
Squamous/non-squamous	33/67	48.6/51.4	49.2/50.8	43.1/56.9	36.8/63.2
MPR	52	36.9	27.7	30.2	52
pCR	41	24	17.2	18.1	37
**Survival (%)**					
Event-Free Survival at 24 months	76	63.8	63.3	62.4	67.2
Overall survival at 24 months	93	82.7	-	80.9	85

## Data Availability

The data that support the findings of this study are not publicly available due to the sensitive and confidential nature of the data, which includes patient information. Access to the de-identified data set may be granted upon reasonable request to the corresponding author and with the approval of the University of Miami Institutional Review Board, in compliance with institutional and ethical guidelines.
